# SAP-Net: A Simple and Robust 3D Point Cloud Registration Network Based on Local Shape Features

**DOI:** 10.3390/s21217177

**Published:** 2021-10-28

**Authors:** Jinlong Li, Yuntao Li, Jiang Long, Yu Zhang, Xiaorong Gao

**Affiliations:** School of Physical Science and Technology, Southwest Jiaotong University, Chengdu 610031, China; YTli@my.swjtu.edu.cn (Y.L.); jianglong@my.swjtu.edu.cn (J.L.); zhangyu.er@home.swjtu.edu.cn (Y.Z.); gxr@swjtu.edu.cn (X.G.)

**Keywords:** point cloud, registration, deep learning, feature extraction, robustness

## Abstract

Point cloud registration is a key step in the reconstruction of 3D data models. The traditional ICP registration algorithm depends on the initial position of the point cloud. Otherwise, it may get trapped into local optima. In addition, the registration method based on the feature learning of PointNet cannot directly or effectively extract local features. To solve these two problems, this paper proposes SAP-Net, inspired by CorsNet and PointNet++, as an optimized CorsNet. To be more specific, SAP-Net firstly uses the set abstraction layer in PointNet++ as the feature extraction layer and then combines the global features with the initial template point cloud. Finally, PointNet is used as the transform prediction layer to obtain the six parameters required for point cloud registration directly, namely the rotation matrix and the translation vector. Experiments on the ModelNet40 dataset and real data show that SAP-Net not only outperforms ICP and CorsNet on both seen and unseen categories of the point cloud but also has stronger robustness.

## 1. Introduction

The 3D point cloud data have incomparable advantages over 2D images, which can accurately record the 3D shape, geometric size, space coordinates, and other information of the object surface. In point cloud data processing, registration is one of the most important tasks, which directly affects the result of model reconstruction. Therefore, point cloud registration also holds great potential in a number of engineering applications including robotics [1], autopilot [2], SLAM [3], and railway transportation [4]. It plays an important role in the handling of component failures in railroad systems, 3D splicing, and other intermediate processes. At present, the iterative closest point (ICP) algorithm [5] is the most extensively used and classic fine registration method with both strong accuracy and versatility. However, ICP also has great limitations. For example, it takes a long time, and it may get trapped into local optima. To optimize the performance of registration, various algorithms based on ICP have been proposed, such as generalized-ICP [6], multi-channel generalized-ICP [7], and sparse ICP [8]. However, these methods still have fundamental drawbacks; they are still sensitive to the initial conditions of the point cloud and are more time-consuming than the original ICP method; the go-ICP [9] method alleviates some drawbacks (such as easily falling into local optimum) but the cost of the algorithm is significantly higher. It can be found that the normal distribution transform (NDT) based on probability distribution uses the matrix method to solve the point cloud matching [10]. A probability model based on multiple Gaussian mixture models (GMM) [11] is used for registration among multiple point clouds. The coherent point drift (CPD) algorithm [12] can effectively complete the registration (albeit with a long computing time). The above probability-based methods usually need to process and transform point clouds, and are difficult to apply to data with diverse shapes. In addition, random sample consensus (RANSAC) based on eliminating mismatched points is also a common method for point cloud registration [13]. Although this improves the outliers in registration, it cannot show better adaptability under complex interference. In view of the problems existing in various traditional algorithms, learning-based registration methods have gradually become a hot spot in recent years. Deep learning networks based on a large number of multi-class data training have improved the accuracy and generalization of registration tasks, which is incomparable to traditional methods.

PointNet [14] uses a multi-layer perceptron to extract features from the point cloud, and then uses a deep learning network to build global features to achieve different tasks such as classification, semantic segmentation, and partial segmentation. It takes the point cloud data as the inputs for the first time to achieve point cloud recognition and end-to-end point cloud processing. To obtain local features and process point clouds effectively, many methods have been proposed including PointNet++ [15], PointCNN [16], and DGCNN [17]. In terms of point cloud registration, PointNetLK [18] uses the PointNet network to extract features and then adjusts the Lucas and Kanade algorithm [19] to successfully achieve the registration, and the network also supports unseen point cloud models to complete the registration. DCP [20], RPM-Net [21,22], and CorsNet [23] can be used to achieve higher accuracy for the registration results of seen and unseen categories. However, these methods rely on inputs with unique local geometric features to predict reliable feature point matching, so they are more sensitive to noise and other interference.

CorsNet architecture can be seen as two parts, namely, the global feature extraction and the correspondence estimation. CorsNet uses PointNet to extract features and then combines point features with global features according to the principle of the PointNet network to obtain more effective registration information. Then, CorsNet uses singular value decomposition (SVD) to estimate the final rigid transformation. It can be understood from PointNet++ that the point features extracted by PointNet cannot represent local features. Therefore, when concatenating global features with them, the information obtained by CorsNet is inaccurate since local shape features are not taken into account. The set abstraction layer of PointNet++ can sample local points layer by layer and transfer features, effectively taking into account the local features of the point cloud. Therefore, if the principle of PointNet++ is used to select features for CorsNet, the features are more accurate and the information obtained is more effective.

In this paper, we propose an end-to-end point cloud registration network, based on deep learning, called SAP-Net. Inspired by CorsNet and PointNet++, SAP-Net is classified into a feature extraction layer (set abstraction (SA)) and a transform prediction layer. Unlike CorsNet, SAP-Net uses PointNet to directly output the six parameters of the point cloud registration in the transform prediction layer to obtain the rigid transformation of the registration. Therefore, SAP-Net can be seen as an optimized CorsNet. We trained our network and CorsNet on the ModelNet40 dataset [24], and the experimental results show that SAP-Net not only outperforms the traditional ICP algorithm but also is better overall than CorsNet. More importantly, SAP-Net has stronger learning ability and better robustness.

The main contributions of this paper are summarized as follows:

1. As the optimization and upgrade of CorsNet, we used the SA layer in PointNet++ as the feature extraction layer, which has been applied in a point cloud registration network directly, and we directly obtained the most effective registration information by connecting the global feature and the initial template point cloud, including the information fusion of Euclidean space and feature space which CorsNet lacks.
2. Unlike the fully connected and SVD methods, we used the PointNet structure as the transform prediction layer to obtain the rigid transformation directly, which reduced the complexity of the network and effectively utilized the local shape features and global features of two point clouds;
3. We compared the proposed method with other methods and evaluated them. Experiments on the general dataset and real data show that this method can be adopted to obtain more effective information, and has stronger learning ability and robustness.

The remainder of this paper is as follows. Section 2 describes the main problems of point cloud registration. Section 3 introduces the point cloud registration of the local feature extraction network based on PointNet++ and transformation output network based on PointNet, as well as the loss function of network training. Section 4 provides the experimental evaluation results. Finally, Section 5 represents the conclusions of this paper.

## 2. Problem Statement

In this section, we will discuss how to obtain the rigid transformation in point cloud registration. We use $P_{S}$ and $P_{T}$ to denote the source point cloud and template point cloud, respectively, where $P_{S}:X=\left\{x_{1},x_{2},\cdots ,x_{n}\right\}\subset R^{3}$ and $P_{T}:Y=\left\{y_{1},y_{2},\cdots ,y_{n}\right\}\subset R^{3}$. When dealing with the point cloud registration problem, we need to find the rigid transformation M∈SE(3), which includes the alignment between $P_{S}$ and $P_{T}$. The transform M is represented as follows:

(1) $$M=\left[\begin{array}{cc} R & T\\ 0 & 1 \end{array}\right]$$

where R∈SO(3) denotes the rotation matrix and $T\in R^{3}$ denotes the translation vector. The registration problem can be defined as

(2) Y=RX+T

The rotation matrix with angle α around the x-axis can be defined as

(3) $$R_{x}=\left[\begin{array}{ccc} 1 & 0 & 0\\ 0 & \cos\alpha & -\sin\alpha \\ 0 & \sin\alpha & \cos\alpha \end{array}\right]$$

Similarly, the rotation matrix with angle β around the *y*-axis and the rotation matrix with angle γ around the *z*-axis can be defined as

(4) $$R_{y}=\left[\begin{array}{ccc} \cos\beta & 0 & \sin\beta \\ 0 & 1 & 0\\ -\sin\beta & 0 & \cos\beta \end{array}\right]$$

(5) $$R_{z}=\left[\begin{array}{ccc} \cos\gamma & -\sin\gamma & 0\\ \sin\gamma & \cos\gamma & 0\\ 0 & 0 & 1 \end{array}\right]$$

So, the rotation matrix R can be represented as follows:

(6) $$R=R_{x}\cdot R_{y}\cdot R_{z}$$

And the translation vector T can be defined as

(7) $$T=\left[\begin{array}{c} t_{x}\\ t_{y}\\ t_{z} \end{array}\right]$$

Finally, it can be found that we only need to solve the six parameters, which is ${\left[\alpha ,\beta ,\gamma ,t_{x},t_{y},t_{z}\right]}^{T}$, and then we can get the rigid transformation of the point cloud registration.

## 3. Method

### 3.1. Network Architecture

**Figure 1:**
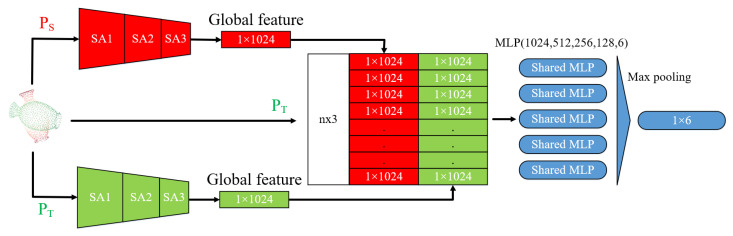
SAP-Net architecture. It consists of two parts: a feature extraction layer and transform prediction layer. SAP-Net uses the SA layer in PointNet++ as the feature extraction layer and then connects the global features and the initial template point cloud. Finally, it uses the PointNet structure as the transform prediction layer to obtain the rigid transformation.

In this section, we will give a brief description of the proposed network structure in Figure 1. The model mainly consists of two parts, namely feature extraction layer and transform prediction layer. In short, we use the SA layer as the feature extraction layer and take PointNet as the transform prediction layer to directly output the six parameters of the point cloud registration, which represent the rigid transformation.

### 3.2. Feature Extraction Layer

We used the SA layer in PointNet++ as the feature extraction layer to extract the features of two point clouds respectively. Compared with the PointNet used in CorsNet, a SA layer can learn hierarchical features, learn the local features of the point, and transfer them layer by layer. As a result, the final global features are more accurate.

In general, the goal of each SA feature extraction layer is to continuously extract local features and expand the local range using the basic principles of down-sampling and PointNet’s high-dimensional feature mapping to obtain a global set of features. Specifically, a SA layer takes the point cloud with n points, and each point $p_{i}=\{x_{i},f_{i}|i=1,2,\ldots ,n\}$ covers its XYZ coordinates $x_{i}$ and its feature $f_{i}$. The layer firstly samples $n^{\prime }$ regions from the input points. These regions are generated based on the sampling points $p_{j}^{\prime }$ of a point cloud determined by the farthest point sampling, and $n^{\prime }$ spherical neighborhoods are generated with these sampling points as the center of mass, where the spatial distance between the neighborhood points in the spherical neighborhood and the center of mass represents the local information. Then a down-sampled point cloud with $n^{\prime }$ points is output, and each point $p_{j}^{\prime }=\{x_{j}^{\prime },f_{j}^{\prime }|j=1,2,\ldots ,n^{\prime }\}$ covers its XYZ coordinates $x_{j}^{\prime }$ and its feature $f_{j}^{\prime }$. These local features will be further extracted and pooled by iterative aggregation. Therefore, each SA layer extracts its local feature with the following symmetric function in each sampled region (defined by a neighborhood specified by radius r):

(8) $$f_{j}^{\prime }=\underset{\left\{i\;|\;\|x_{i}-x_{j}^{\prime }\|\leq r\right\}}{Max}\left\{h\left(f_{i},x_{i}-x_{j}^{\prime }\right)\right\}$$

where h denotes the multi-layer perceptron (MLP), Max denotes the max pooling.

The third layer SA module no longer performs sampling and local area generation, but aggregates high-dimensional features to obtain the global feature information of the target. Compared with the PointNet-type feature aggregation network similar to CorsNet, our feature extractor can learn higher-level features containing metric spatial distance information that are getting larger and larger at the local scale through the expansion of each layer’s neighborhood. This enhances the ability to extract the shape and structure information of the point cloud, and provides global features containing rich local features for point cloud registration. In addition, the radius range is increased layer by layer by setting the spherical neighborhood and the number of sampling points of each SA layer, so that the neighborhood features are continuously expanded. We explained the specific settings of the SA layer in the experimental section.

### 3.3. Transform Prediction Layer

After obtaining the global feature of the source point cloud and template point cloud, Corsnet combines the 64-dimensional feature representing local information of the source point cloud with the 1024-dimensional feature of two point clouds as the basis for the computing transformation. However, this direct concatenation of different deep features of PointNet does not adequately represent the local features of the point cloud and also contains only high-dimensional information in the feature space. In this paper, SAP-Net fed the global feature back to $P_{T}$ by concatenating the 1024-dimensional feature of two point clouds with the template point cloud. The extracted global feature is connected to the coordinates of each point of the point cloud to be aligned, which contains the local feature information and Euclidean spatial information of the point cloud. It is a full consideration of the local shape and relative position of the two point clouds. The amount of data in three-dimensional coordinates is also more economical than the 64-dimensional feature selected by CorsNet. Therefore, this type of feedback can directly find the differential information between two point clouds, which is more useful for the registration.

Furthermore, the final output of CorsNet is a n×3 matrix, and then SVD is used to calculate the rotation matrix and the translation matrix. However, SVD requires more accurate prediction of matching point pairs. The global features aggregated by the PointNet principle cannot represent the unique geometric structure of each layer, which makes learning matching point pairs very difficult. Instead, in this paper, SAP-Net used the PointNet as the transform prediction layer, namely, MLP and max pooling. This is because the global features extracted by SAP-Net are the aggregation of local features, and they learn the geometric knowledge of the entire point cloud. It is more suitable to use the parameter learning ability of the deep learning network to directly predict the transformation matrix required for registration. Finally, SAP-Net outputs a 1 × 6 vector, which is the six parameters ${\left[\alpha ,\beta ,\gamma ,t_{x},t_{y},t_{z}\right]}^{T}$.

### 3.4. Loss Function

Since only six parameters of the rotation matrix and the translation vector are needed for the point cloud registration problem in this paper, the goal of our loss function is to make the transformation of point cloud rotation and translation closer to the real transformation, and choose a simple and effective error measure. To constrain and reduce the difference between the predicted value and the truth value, the loss function is defined as

(9) $$Loss={\left\|R^{T}R^{g}-I\right\|}^{2}+{\left\|t-t^{g}\right\|}^{2}$$

where R denotes the rotation matrix, t denotes the translation vector, g denotes the ground truth, and T denotes the matrix transpose. 

## 4. Experiments

We experimented on the ModelNet40 dataset, which covered 12,311 3D CAD models from 40 categories. ModelNet40 dataset is one of the most commonly used datasets as a benchmark for testing point cloud registration methods, with sufficient sample and various types, so we evaluate our experimental results according to this dataset. We used 9843 models as the training set and 2468 models as the test set, where the ratio was close to 4:1. Like PointNet, 1024 points were uniformly sampled from the surface of each model as an initial point cloud, the points were centered and only XYZ coordinates were used as input.

For convenience, we denote + as the combination of the feature extraction layer and the transform prediction layer, PN as the PointNet, FC as the full connection layer, and SVD as the singular value decomposition.

We compared SA+PN (as SAP-Net) with ICP, PN+FC, SA+FC, PN+SVD (as CorsNet), and PN+PN. On the one hand, in the feature extraction layer, the dimensions of each layer in PN are [64, 64, 128, 256, 1024]. Table 1 shows the setup of SA. SA3 is a global set abstraction layer that converts a set to a single vector. On the other hand, in the transform prediction layer, the dimensions of FC (and PN) are [1024, 512, 256, 128, 6].

Adam [25] was used to optimize the network parameters, with an initial learning rate of 0.001. The learning rate was reduced by 10 times at 75, 120, 160, and 200, respectively, for a total of 250 epochs. The experiments with SAP-Net and other approaches were conducted on a computer with Inter i5-10300H CPU, NVIDIA GeForce RTX 2060 GUP, and used the pytorch 1.2 development environment with PyCharm. We measured mean squared error (MSE), root mean squared error (RMSE), and mean absolute error (MAE) between the ground truth values and predicted values. Ideally, the smaller the error metrics are, the more accurate the rigid alignment is.

### 4.1. Train and Test on ModelNet40

First, we randomly classified all of the point clouds in ModelNet40 into the training set and test set, and different point clouds were used for training and testing. We randomly used a rigid transformation along each axis to generate the template point cloud. The rotation angle was randomly generated in [0, 45] and the initial translation distances were randomly selected in [−0.5, 0.5]. According to this rule, the initial rotation and translation were also performed randomly in the test set.

Table 2 shows the performance of all models. The performance of various methods can be shown through the comparison of various indicators of different methods based on different network layers, and the low error value represents better registration performance.

**Figure 2:**
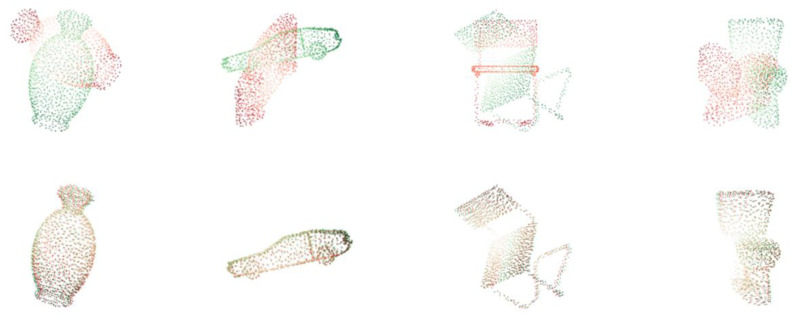
Registration results (red: source point cloud, green: template point cloud). The initial positions of the two point clouds are shown at the top and the results of the alignment are shown at the bottom. It can be seen that the proposed network can also achieve good registration results for methods that may fall into the local optimal solution due to the highly symmetric point cloud.

It can be seen from Table 2 that the ICP registration method which depends on a better initial position is not suitable. SAP-Net outperforms other methods under all the metrics, which is better than the original CorsNet. Figure 2 shows results of SAP-Net on the part of samples in ModelNet40.

**Figure 3:**
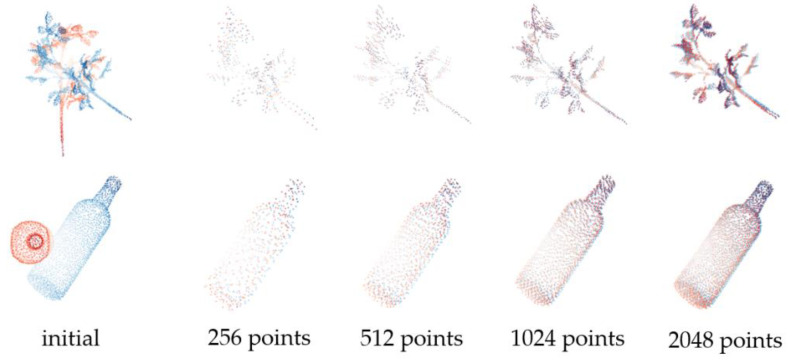
Registration results from sparse to dense sampling.

In addition, to test the generalization of SAP-Net on point clouds with different shape features and sparsity degrees, we selected the sample with simple structure (Bottle) and the sample with more shapes (Plant) to test performance, carried out different degrees of random sampling and the same initial transformation on point cloud samples, and evaluated the mean absolute error of rotation. Table 3 and Figure 3 show the test results.

It can be seen from the results that, except for the accuracy of samples with less points and more missing information, SAP-Net can still maintain good accuracy for point clouds with large shape differences, and has good adaptability to density changes.

### 4.2. Experiment on Different Categories

To test and verify the learning ability of the model, we trained and tested the proposed model on different categories. Under the same conditions, we used the first 20 categories for training and the rest categories for testing. As shown in Table 4, SAP-Net still outperforms all the models on all metrics, which means SAP-Net has a stronger ability of generalization and can learn more useful registration information, which is better than CorsNet.

It can be seen that under the verification condition of clean point cloud data, the three sets of experimental results of PN + FC are even better than PN + SVD of CorsNet but are still far inferior to PN + PN, which is the CorsNet variant. It can be proved that PN, as the transform prediction layer, is more applicable to the network structure of CorsNet. For the feature extraction layer, we used PN and SA for comparison. From the three sets of error experiments, it can be seen that SA + FC is only slightly better than PN + FC. However, SA+PN is better than PN + PN in all metrics, which means that SA as a feature extraction layer is more effective in combination with PN as a transform prediction layer, and so the predicted registration parameters are more accurate.

### 4.3. Robustness Test

In addition, to verify the robustness of the model, we added Gaussian noise to the point cloud for testing. During the training, we use the training method for the full dataset according to the setting in Section 4.1. However, during the testing, we randomly jittered the points in both point clouds by adding Gaussian noise with a mean value of 0 and standard deviation (SD) of 0.01 to each point, clipped the noise to [−0.05, 0.05], and then we added it to the input point cloud.

Table 5 shows the results of the robustness test. PN + FC model is sensitive to noise, and the interference of noise is obvious to the feature extraction layer based on PointNet. SAP-Net still keeps robust to noise and performs best among all the models.

**Figure 4:**
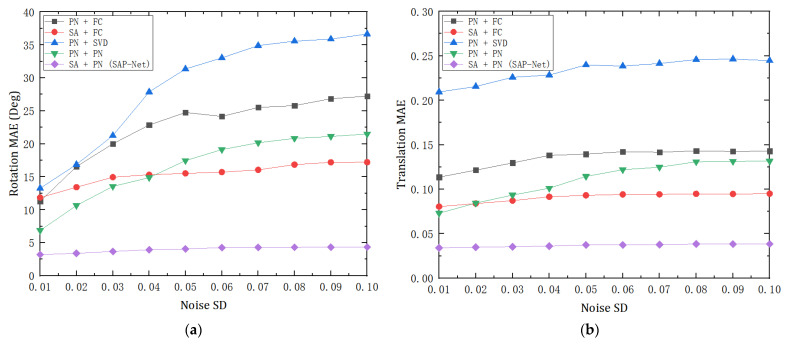
Registration results at different levels of Gaussian noise. (**a**) Mean absolute error (MAE) of rotation and (**b**) mean absolute error (MAE) of translation.

To further verify the robustness of the proposed model in a complex environment, we also compared the performance of various methods under different degrees of noise. So, we used the clean dataset of modelnet40 used in the evaluation in Section 4.1, and randomly added the Gaussian noise of standard deviation (SD) in [0.01, 0.1] with 0.01 as the step size to the two point clouds of each sample to further test the model. Considering the large error of the traditional ICP method, we only compared the learning-based method. As the noise level continued to increase, we also got the mean absolute error (MAE) of rotation and translation in each stage according to the output transformation results, as shown in Figure 4.

Experimental results show that with the increase of noise level, SAP-Net still maintains a stable error result, which is significantly better than other methods in noise impact. In addition, the performance of the SA + FC model is also relatively stable, which is similar to that of SAP-Net, indicating that the SA feature extraction module brings better robustness.

### 4.4. Test on Real Objects

**Figure 5:**
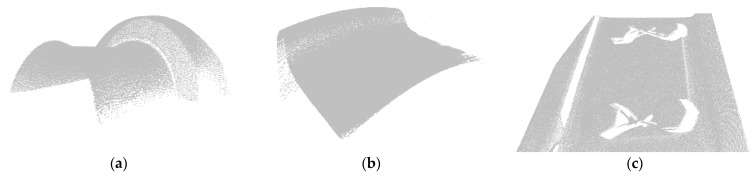
Experimental data of real point cloud in railway transportation. (**a**) Train wheel tread data 1, (**b**) train wheel tread data 2, and (**c**) bolt components.

To verify the applicability of our registration method to different objects, the point cloud data of real objects were tested. This set of experimental data samples is an important three-dimensional object in railway transportation. The real point cloud data are collected by the industrial three-dimensional laser scanning system, and have been preprocessed and can be directly used for algorithm experiments. We tested the train wheel tread and bolt components by weights obtained from the trained model of SAP-Net. The real point cloud data are shown in Figure 5.

In the test process of registration, we still evaluated the general performance of our model in real data through three kinds of error by the calculation of transform predicted value and ground truth. The results of registration accuracy are shown in Table 6 and Table 7, where W1 and W2 represent the two point clouds of the train wheel tread.

**Figure 6:**
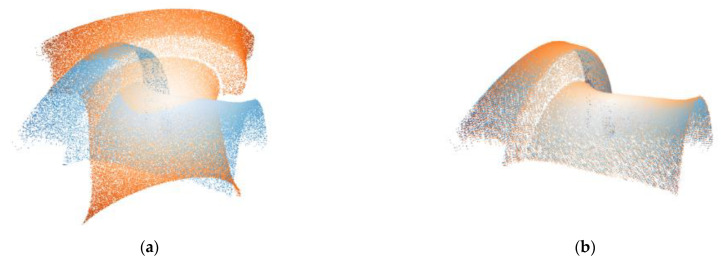

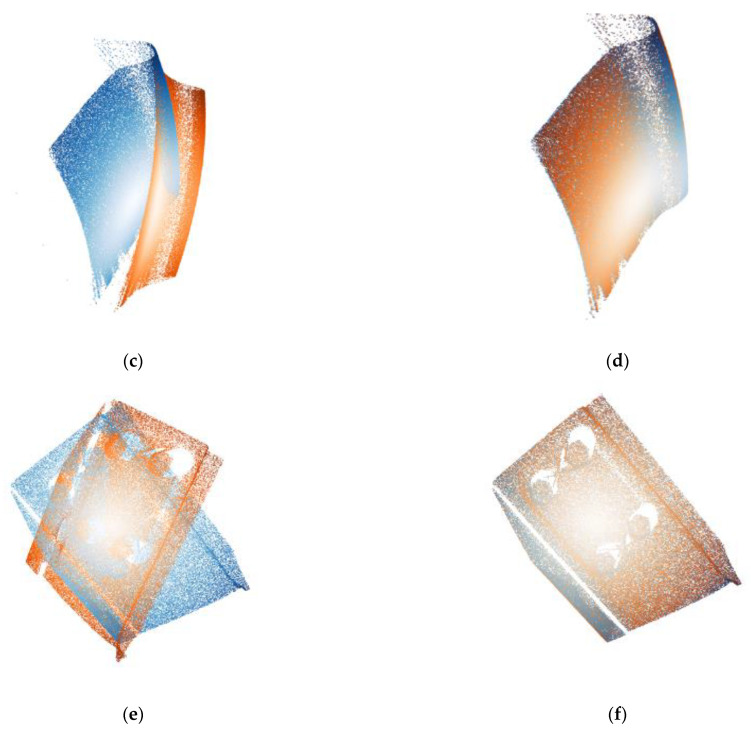
Registration results of real data. (**a**,**c**,**e**) are the initial position of the two point clouds, (**b**,**d**,**f**) are the results of registration, the yellow point set is the source point cloud and the blue point set is the template point cloud.

It can be seen from the experimental data that the trained model based on the common ModelNet40 dataset can still maintain good alignment ability in railway transportation. The experimental results of this group are similar to or better than the results in Section 4.1, showing the usability of the proposed method. Figure 6 shows the alignment results. In addition, for these three point clouds, we compared the average test time (in milliseconds) in this group of experiments, including traditional ICP, learning-based CorsNet, and our SAP-Net, as shown in Table 8. Comprehensive experiments show that our method achieves the expected requirements in the simplicity and stability of the registration network.

## 5. Conclusions

In this paper, based on the CorsNet network structure, we propose a 3D point cloud registration network with a simple structure, called SAP-Net, which firstly uses a set abstraction layer in the network of PointNet++ to extract features and then feed it back to the template point cloud, and finally uses PointNet to predict the rigid transformation. Based on experiments by comparing SAP-Net with ICP, CorsNet, and other variants on the ModelNet40 dataset, we demonstrate and discuss the importance and effectiveness of each part in SAP-Net to prove the accuracy and better robustness of SAP-Net. In some cases of railway transportation, our method also shows good performance. We will try our best to improve the algorithm and network in the point cloud data with more complex scenes, which we regard as our future work.

## Figures and Tables

**Table 1:** Setup of SA ($n^{\prime }$: number of output points; r: the radius defining a neighborhood).

	*n*′	*r*	Dimensions
SA1	512	0.2	64, 64, 128
SA2	256	0.4	128, 128, 256
SA3	/	/	256, 512, 1024

**Table 2:** Test on full dataset. Various implementation combinations are presented by abbreviations (feature extraction + rigid body transformation).

	ICP	PN + FC	SA + FC	PN + SVD	PN + PN	SA + PN (SAP-Net)
MSE (R)	903.497070	47.865341	168.943558	252.624695	37.973038	20.001087
RMSE (R)	30.058228	6.918478	12.997829	15.894172	6.162227	4.472258
MAE (R)	17.923250	5.600912	11.266281	12.779058	4.373697	3.088548
MSE (t)	0.061544	0.019928	0.010722	0.069416	0.002480	0.001695
RMSE (t)	0.248080	0.141168	0.103546	0.263470	0.049800	0.041168
MAE (t)	0.201832	0.122349	0.080985	0.214743	0.039462	0.034388

**Table 3:** Tests at different sampling degrees.

Sampling Points	256	512	1024	2048
MAE (Plant)	3.2606	1.8963	1.1074	1.2551
MAE (Bottle)	2.7916	1.9094	1.0298	0.8435

**Table 4:** Test on different categories.

	ICP	PN + FC	SA + FC	PN + SVD	PN + PN	SA + PN (SAP-Net)
MSE (R)	903.732239	195.343384	181.686539	270.140747	48.905247	22.027050
RMSE (R)	30.062140	13.97653	13.479115	16.435959	6.993228	4.693298
MAE (R)	17.292072	11.461332	11.271105	13.292407	5.008487	3.274244
MSE (t)	0.073674	0.011866	0.004643	0.066987	0.003479	0.002312
RMSE (t)	0.271429	0.108930	0.068141	0.258818	0.058981	0.048084
MAE (t)	0.220805	0.085516	0.053935	0.207364	0.047160	0.040955

**Table 5:** Test with Gaussian noise.

	ICP	PN + FC	SA + FC	PN + SVD	PN + PN	SA + PN (SAP-Net)
MSE (R)	950.946045	169.587784	214.185822	268.276764	78.038536	20.994427
RMSE (R)	30.837414	13.022588	14.635089	16.379156	8.833942	4.581967
MAE (R)	24.432901	11.289206	11.850265	13.264036	6.858354	3.203474
MSE (t)	0.078597	0.020040	0.010414	0.065434	0.007910	0.001673
RMSE (t)	0.280351	0.141563	0.102047	0.255800	0.088937	0.040905
MAE (t)	0.220353	0.113622	0.080460	0.209393	0.073032	0.034165

**Table 6:** Rotation accuracy of real objects in railway transportation.

	MSE (R)	RMSE (R)	MAE (R)
W1	0.606702	0.778911	0.721933
W2	1.659326	1.288148	1.087313
Bolt	1.241919	1.114414	1.089038

**Table 7:** Translation accuracy of real objects in railway transportation.

	MSE (t)	RMSE (t)	MAE (t)
W1	0.007216	0.084946	0.081434
W2	0.006939	0.083303	0.079179
Bolt	0.006825	0.082616	0.079559

**Table 8:** Computational efficiency.

Method	ICP	CorsNet	SAP-Net
Time (ms)	389	104	79

## Data Availability

Not applicable.
